# Waiting List Policy Reform Is Required to Enable Randomised Controlled Trials of Elective Surgery

**DOI:** 10.1111/ans.70744

**Published:** 2026-06-22

**Authors:** Jonathan G. Quicke, Deborah J. Brown, Bernadette Richards, Owen Ung, Catherine McDougall, Sandra Crone, Nadine E. Foster

**Affiliations:** ^1^ Surgical Treatment and Rehabilitation Service (STARS) Education and Research Alliance The University of Queensland and Metro North Health Brisbane Queensland Australia; ^2^ Centre for Innovation in Pain and Health Research School of Health and Rehabilitation Sciences, The University of Queensland Brisbane Queensland Australia; ^3^ School of Historical and Philosophical Inquiry, and Centre for Innovation in Pain and Health Research (CIPHeR) University of Queensland Brisbane Australia; ^4^ Medical School University of Queensland Herston Queensland Australia; ^5^ Breast and Endocrine Surgery Comprehensive Breast Cancer Institute (CBCI), Royal Brisbane and Women's Hospital, Metro North Health Herston Queensland Australia; ^6^ Queensland Health Brisbane Queensland Australia; ^7^ Consumer Co‐Author Partner, Australia New Zealand Musculoskeletal Clinical Trials Network (ANZMUSC) Consumer Advisory Group Melbourne Victoria Australia

**Keywords:** elective surgery, ethics, law, policy, randomised controlled trials, surgery, waiting lists

## 1 The Waiting List Challenge for Trials of Elective Surgery

Randomised controlled trials (RCTs) are the gold standard for evidencing the comparative clinical and cost effectiveness of elective surgery [1, 2]. However, only around 1 in 5 surgical RCTs recruit to time and target [3]. Potential solutions to better‐enable surgical RCTs include ring‐fenced research resources, allocated trial capacity, more streamlined governance processes and registry based RCTs [4]. Here we address another key factor jeopardising successful RCT recruitment in Australia—the impact of waiting lists [4], with the legacy system favouring adherence to waiting lists irrespective of RCTs. We challenge the prevailing assumption that it is unfair to prioritise participants in elective surgery trials over medically similar non‐participating patients, arguing that this rests on an incomplete understanding of the nature of fairness.

Current regulation (both broad policy and guidelines) of elective surgical waiting lists and whom should be prioritised is silent on RCTs (Box 1). This leaves surgical services engaged in RCTs to either (1) treat RCT participants the same as non‐participating patients, or (2) apply their own prioritisation criteria, potentially circumventing national/state/local directives. Both ‘solutions’ are ethically problematic and we advocate for a third option: developing policy that warrants prioritising RCT participants. We justify this proposal by contrasting 2 scenarios (Box 2) relating to a hypothetical RCT investigating the clinical and cost effectiveness of total knee replacement (TKR) compared to conservative care. National median waitlist time for elective TKR surgery, is 265 days in the public sector [5].

BOX 1 **Current legislation, guidelines and policy covering surgical waiting list practice.**

**Table:** 

State/territory	Legislation/ policy	Resources available at
Australia	National elective surgery urgency categorisation 2015	https://www.scribd.com/document/796896706/National-Elective-Surgery-Categorisation-1
Australian capital territory	Canberra health services policy elective surgery access [ACT Government]	https://www.canberrahealthservices.act.gov.au/about-us/policies-and-guidelines
New South Wales	Planned surgery access	https://www1.health.nsw.gov.au/pds/ActivePDSDocuments/PD2025_036.pdf
Queensland	Elective Surgery Services Implementation Standard [Qld government]	https://www.health.qld.gov.au/__data/assets/pdf_file/0027/397440/qh-imp-342-1.pdf
South Australia	Elective surgery access and management policy [Government of South Australia]	https://www.sahealth.sa.gov.au/wps/wcm/connect/public+content/sa+health+internet/about+us/governance/policy+governance/policies/elective+surgery+access+and+management+policy
Tasmania	Planned surgery access policy and operational standards 2024 [Tasmanian Government]	https://www.health.tas.gov.au/sites/default/files/2025-07/DOH10222PlannedSurgeryAccessPolicy20Web_AA.pdf
Victoria	Planned surgery access policy 2024 [Victoria State Government]	https://www.health.vic.gov.au/surgical-services/planned-surgery-access-policy-2024
Western Australia	Elective services access standard & elective services access and management policy [Government of Western Australia]	https://www.health.wa.gov.au/~/media/Corp/Policy-Frameworks/Clinical-Services-Planning-and-Programs/ Elective-services-access-and-management-policy/supporting/Elective-Services-Access-Standard.pdf https://www.health.wa.gov.au/~/media/Corp/Policy-Frameworks/Clinical-Services-Planning-and-Programs/ Elective-services-access-and-management-policy/Elective-Services-Access-and-Management-Policy.pdf

BOX 2 **Two scenarios exploring waiting list practice in surgical RCTs.**

**Scenario 1: RCT participants and non‐participating patients are treated the same.**

All medically similar patients awaiting TKR surgery (in our example, non‐urgent elective surgery patients) are waitlisted and whether they choose to take part in the RCT makes no difference to their position on the list. This practice is justified on grounds of fairness— that is, ‘equality of care for all based on their clinical need’ or Strict Egalitarianism—which is argued aligns with a key goal of the National Health Reform Agreement (NHRA) to ‘establish a fair and sustainable healthcare system nationwide’ [10].

**Scenario 2: RCT participants are prioritised over non‐participating patients.**

All medically similar patients awaiting TKR surgery are waitlisted. Those who agree to take part in the RCT are prioritised with the result that some patients listed for surgery have to wait longer than the median time for their surgery. This practice is justified on the grounds that it will increase the likelihood of meaningful evidence generation and ultimately secure greater future benefits for patients, surgical services, healthcare systems and society. This too aligns with ‘driving best‐practice using data and research’, another NHRA goal [10]. It is noted that this prioritisation does not supersede the timely provision of surgical care for medically urgent cases.

Our proposal raises an ethical dilemma requiring the balancing of different interests. Scenario 1 upholds the principle of justice through equal access to healthcare. However, if this is reconsidered by instead prioritising those enrolled in the RCT—Scenario 2—, then the broader public good is maximised through generation of best evidence.

In Scenario 1, waiting lists delay or prevent RCT recruitment (which is limited by finite funding and a fixed end date), lead to an underpowered RCT, increased chance of Type II error, and ongoing clinical uncertainty [6]. This indirectly harms future patients, surgeons, healthcare systems, researchers and taxpayers who fund RCTs through government funding bodies like National Health Medical Research Council (NHMRC) and the Medical Research Future Fund (MRFF) scheme. All these interest holders may ultimately become disillusioned with the feasibility and value of surgical RCTs.

From a utilitarian perspective, Scenario 2 is well‐justified. By removing long delays for elective surgery RCT participants, the trial has a higher chance of recruiting its target sample size and providing precise estimates of clinical effectiveness and cost‐effectiveness. However, we are mindful that in Scenario 2, somebody further down the list ‘jumps the queue’—and could that be considered unfair? Since fairness is a fundamental principle of a just healthcare system (NHMRC National statement), we now make the case that Scenario 2 can be justified as being *fairer* than Scenario 1.

### 1.1 Fairness Argument

We draw on ethical arguments from the World Health Organisation during the COVID‐19 pandemic [7] to show that fairness does not demand strict egalitarianism but requires other factors be brought to bear on decisions, including patient choice, social benefit and best research evidence generation (Box 3).

BOX 3 **Fairness beyond strict egalitarianism.**

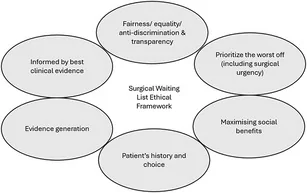

Adapted from Saadeh et al. Frontiers in Medicine 2021 [11].

Rationing in healthcare services is inevitable. Waitlists offer one way of dealing with this reality, but some flexibility in how waitlists are managed is necessary. If Patient 19 forgoes the chance of surgery next Monday because they have tickets to the football, it would be unreasonable to hold off the surgery until 19 is available rather than offer it to Patient 20. Egalitarianism does not dictate that people should be subsidised for their choices—people are expected to pay the true social cost of their choices [8]. Is it so different if Patient 19 chooses not to participate in an RCT and so misses out on the surgery next Monday, which is offered instead to Patient 20 who agrees to participate (and who takes on risks and burdens of participation)?

For policy decisions to be guided by the principle of fairness, it is also necessary to take into consideration what is fair to all those who are affected by the decision [9]. Here, this encompasses society broadly including current and future patients, health services, surgeons and taxpayers, all of whom stand to benefit from the RCT results and cannot reasonably be expected to subsidise the choices of non‐participants.

It is generally thought that some inequalities must always be justifiable as fair—for example, unequal distributions of resources that benefit the worst‐off or promote equity [12]. It is not unfair to feed aid workers first so that they can distribute food to starving people. Similarly, it is not unfair to treat first volunteers in an RCT designed to reduce uncertainty about surgical treatments that stand to benefit all future patients. One key caveat would be the concomitant need to improve equitable access to RCTs to avoid exacerbating existing inequities among those too often excluded from RCTs (e.g., residents in rural/remote areas, the oldest old, culturally and linguistically diverse groups). This would need to be considered carefully and addressed in any future waiting list policy reform.

### 1.2 Legal Implications and the Coercion Debate

Informed and voluntary consent to participate in an RCT is a legal and ethical requirement [13]. To ensure ‘system level fairness’, all patients at the point of being admitted to the waitlist should be informed of the hospital's commitment to research and prioritisation practices. That deals with the ‘informed’ part but more worrisome is how to ensure that consent remains voluntary.

Evaluating whether our proposal is potentially coercive is complex. Practically, the potential for coercion is greater in systems that allow unchecked recruitment incentives. Strict regulations and, ideally, a federal regulatory body, are needed to mitigate against hospitals and clinicians being incentivised to recruit participants to RCTs, especially by industry sponsors [14]. Next, there is the question of whether patients would be incentivised to participate in an RCT solely by the promise of moving up the waitlist. Any assumption that this is obvious would need to be tempered by consideration of the disincentives—the risks and burdens of participation. There is no categorical answer to the question whether prioritising participants for an RCT is potentially coercive or not. It depends on the number of participants sought and what the significance (threat) of that is to those on the waitlist who decline—that is, how far down a waitlist a patient should reasonably be expected to drop for the wider RCT benefits for society. All of this would need to be communicated clearly in the RCT recruitment process and considered on a case‐by‐case basis to determine whether deviation from the status quo waitlist approach is justified.

## 2 Conclusion

To better evidence elective surgical care, we recommend policy review at a national and state level to allow participants in elective surgical RCTs to be prioritised and progress on waiting lists to better ensure publicly funded RCTs recruit to time and target. However, to justify any move from ‘perspective to policy’, we recommend broader consultation and welcome ongoing ethical debate. We plan to conduct participatory action research and discrete choice experiments including surgeons, patients and interest holders comparing the opposing principles and scenarios discussed in this article. This will help to build the evidence base for improved policy and decision making in the future.

## Author Contributions

As lead author for all aspects of this work including conception and leading the drafting and critical revision of the manuscript, JQ is responsible for the overall integrity of the work. DB, BR, OU, CMcD and NF were all involved in the conceptualisation, drafting of the manuscript, and revising it critically for important intellectual content. SC was involved in the drafting of the manuscript and revising it critically for important intellectual content. All authors approved the final manuscript.

## Funding

Dr Jonathan Quicke is supported through a National Health and Medical Research Council (NHMRC) Investigator Grant Leadership 2 (#2018182) awarded to Prof Nadine Foster. Prof NE Foster is supported by a National Health and Medical Research Council (NHMRC) Investigator Grant Leadership 2 (#2018182). The views expressed are those of the authors.

## Conflicts of Interest

The authors declare no conflicts of interest.

## Data Availability

Data sharing not applicable to this article as no datasets were generated or analysed during the current study.
